# Near-Infrared Light Photodynamic Therapy with PEI-Capped Up-Conversion Nanoparticles and Chlorin e6 Induces Apoptosis of Oral Cancer Cells

**DOI:** 10.3390/jfb15110333

**Published:** 2024-11-07

**Authors:** Jinhao Cui, Yoshimasa Makita, Tomoharu Okamura, Chihoko Ikeda, Shin-ichi Fujiwara, Kazuya Tominaga

**Affiliations:** 1 Department of Oral Pathology, Osaka Dental University, 8-1 Kuzuhahanazonocho, Hirakata 573-1121, Osaka, Japan; okamu-t@cc.osaka-dent.ac.jp (T.O.); ikeda-c@cc.osaka-dent.ac.jp (C.I.); tominaga@cc.osaka-dent.ac.jp (K.T.); 2 Department of Chemistry, Osaka Dental University, 8-1 Kuzuhahanazonocho, Hirakata 573-1121, Osaka, Japan; makita@cc.osaka-dent.ac.jp (Y.M.); fujiwara@cc.osaka-dent.ac.jp (S.-i.F.)

**Keywords:** NIR-PDT, oral squamous cell carcinoma cells, apoptosis, p53

## Abstract

Oral squamous cell carcinoma (OSCC) is a common malignancy in the oral cavity. Photodynamic therapy (PDT) is a new alternative for the treatment of diseases using photosensitizers (PS) and light. In this study, we used a photosensitizer complex (Ce6-MnNPs—Chlorin e6 combined with up-conversion nanoparticles NaYF_4_:Yb/Er/Mn) to investigate the therapeutic effectiveness of this treatment against oral cancer cells. We also investigated the mechanism of action of near-infrared light PDT (NIR-PDT) combined with the Ce6-MnNPs. After determining a suitable concentration of Ce6-MnNPs using an MTT assay, human oral squamous cell carcinoma cells (HSC-3) were treated with NIR-PDT with Ce6-MnNPs. We examined the characteristics of Ce6-MnNPs by transmission electron microscopy (TEM); a zeta potential and particle size analyzer; Fourier-transform infrared spectroscopy (FTIR); cell viability by MTT assay; and apoptosis by FITC-Annexin V/PI assay. The mitochondrial membrane potential (MMP), apoptosis-related mRNA level (Bax and Bcl-2) and p53 protein were also researched. NIR-PDT with 0.5 ng/µL Ce6-MnNPs inhibited the proliferation of HSC-3 (*p* < 0.05). After treatment with NIR-PDT, changes in the mitochondrial membrane potential and apoptosis occurred (*p* < 0.01). The ratio of Bax/Bcl-2 and p53-positive cells increased (*p* < 0.01). These results suggest that this treatment can induce apoptosis of oral cancer cells.

## 1. Introduction

Oral cancer is usually a malignant tumor that occurs in the lips, cheeks, gingivae or tongue, with more than 377,700 cases worldwide in 2020 [1]. Most of the oral tissues are covered with squamous epithelium; so 90% of oral cancer is squamous cell carcinoma (OSCC). OSCC disrupts the patient’s speech, feeding and other functions [2,3]. Current treatment is based on surgery combined with radiotherapy and chemotherapy, and post-treatment comorbidities have a long-term impact on the patient’s quality of life [4,5].

The underlying reaction was discovered by Oscar Raab and Professor Herman von Tappeiner in 1900 in Germany, and the term photodynamic reaction was introduced in 1907 [6]. Photodynamic therapy (PDT) is used today for the treatment of diseases in a variety of fields. In the field of dentistry, it can be used for systemic treatment in the form of blood administration or conservative treatment for precancerous lesions and carcinoma in situ, or it can be used in combination with chemotherapy and radiotherapy to enhance the efficacy [7]. According to various studies, it has been reported that photosensitizer complexes coated with polyglycerol and loaded with adriamycin and chlorin e6 (Ce6) show effectiveness against melanoma, and ala-PDT is effective in the systemic treatment of malignant diseases of the oral cavity and in local application in carcinoma in situ [7,8]. In addition to visible light with a wavelength of about 600 nm, light sources such as near-infrared light (NIR) have been widely studied and applied in recent years [9]. Photosensitizers (PS) are also evolving. Today’s PS have better targeting properties and longer absorption wavelengths and are optimized when combined with antibodies or amino acids, or encapsulated in nanoparticles [10]. The preferential accumulation of photosensitizers in tumor tissue is due to the loose epithelial cells of tumor tissue, which lack cell-lined tumor vasculature [11]. Ce6, a widely used second-generation PS, is inherently poorly water-soluble and is not easily translocated to the tumor site when used alone, which has prompted a variety of techniques to improve efficacy [12]. In this study, the light source used was NIR at 980 nm so that deeper tissue layers could be reached [13]. Nanoparticle photosensitizer complexes were also synthesized in order to overcome the problems of low energy conversion efficiency and the high degree of hydrophobicity. NaYF_4_:Yb/Er is a typical up-conversion nanoparticle that can convert long-wave radiation to short-wave radiation. The incorporation of manganese ions (Mn^2+^) attenuates the anti-PDT ability conferred by glutathione to cancer cells. Encapsulation of polyethyleneimine (PEI) improves stability under physiological conditions [14,15].

The type of cell death caused by PDT depends on the type and intracellular localization of the PS, as well as the intensity of the light exposure [16,17]. Apoptosis is the main type of cell death after PDT. It is programmed cell death and divided into early and late apoptosis [18]. Mitochondria are involved in one of the main processes of apoptosis, including the release of cytochrome c, caspase-3, -6, and the expression of apoptosis-related proteins, such as pro-apoptotic proteins Bax and anti-apoptotic proteins Bcl-XL, Bcl-w in the Bcl-2 family [18,19].

p53, a tumor suppressor, is expressed at low levels in normal conditions. After its activation by cellular stress, it transactivates some target genes that induce cell cycle arrest and apoptosis. Therefore, in tumor therapy, correctly inducing p53 activation is also a way to improve therapeutic efficacy [20,21,22]. However, there are fewer studies on photodynamic therapy to upregulate oncogene expression in the treatment of oral cancer.

In this study, we utilized a synthesized nanoparticle photosensitizer complex combined with near-infrared light PDT (NIR-PDT) to observe its effect on human tongue squamous cell carcinoma cells (HSC-3) and induction of apoptosis and expression of p53.

## 2. Materials and Methods

### 2.1. Chemical Reagent Preparation

Chemical reagents required for synthesis are shown in Table 1.

### 2.2. Synthesis of PEI-Capped Nanoparticles/Ce6

The PEI-capped nanoparticles/Ce6 were synthesized according to a paper by Kamimura et al. [23]. YCl_3_•H_2_O (146 mg, 480 µmol), YbCl_3_•6H_2_O (41.9 mg, 108 µmol), ErCl_3_•6H_2_O (4.6 mg, 12 µmol) and MnCl_2_•4H_2_O (156 mg, 790 µmol) were added to 0.6 mL of distilled water. The mixture was added to 17 mL of EG solution containing NaCl (134 mg, 2.3 mmol) and PEI (306 mg, 0.17 mmol). Then, the mixture was stirred at room temperature for 60 min. A total of 11 mL of EG solution containing NH_4_F (174 mg, 4.7 mmol) was added to the mixture. The reaction mixture was poured into a PTFE-lined autoclave (Huanyu, Jiangsu, China) and heated at 200 °C for 6 h. After cooling to room temperature, the obtained nanoparticles were purified by high-speed centrifugation (20,000× *g*, 20 min, ×3) (Hitachi, Tokyo, Japan) and washed with ethanol. Ce6 (30 mg, 0.017 mmol), NHS (17.4 mg, 0.05 mmol) and 1-ethyl-3-(3-dimethylaminopropyl) carbodiimide (EDC) (31.8 mg, 0.055 mmol) were dissolved into 1 mL of N, N-dimethylformamide (DMF). The solution and the obtained nanoparticles were co-dissolved into 10 mL of ultrapure water, and the pH was adjusted to 5.0 with hydrogen chloride (HCl) and sodium hydroxide (NaOH). The reaction mixture was left at room temperature for 24 h and then purified by centrifugation (20,000× *g*, 10 min, ×2) and washed with ethanol. After removing the supernatant by decantation, the obtained nanoparticles were dried in vacuo.

### 2.3. Characterization of Synthesized Ce6-MnNPs

The Ce6-MnNPs’ size and zeta potential were measured with a transmission electron microscope (TEM: H-7100; Hitachi, Tokyo, Japan) and a zeta potential and particle size analyzer (ELSZ-2000, Otsuka Electronics, Osaka, Japan). A Fourier-transform infrared spectroscopy (FT-IR) spectrophotometer (IRAffinity-1S, Shimadzu, Kyoto, Japan) was used to investigate the bonds and functional groups present and shifts in Ce6, PEI and Ce6-MnNPs. The data were analyzed using OriginPro 2024 software (OriginLab, Northampton, MA, USA).

### 2.4. Measurement of Ce6 Fluorescence

To determine the accumulation of the complex in HSC-3 cells, after its addition to a 96-well plate seeded with cells, the fluorescence intensity of each well was examined after 1, 2, 3 and 4 h using a microplate reader (Molecular Devices, San Jose, CA, USA) at Ex 450 nm and Em 650 nm [24].

### 2.5. Determination of Suitable Treatment Conditions (MTT Assay)

The HSC-3 cell line was provided by the Second Department of Oral and Maxillofacial Surgery, Osaka Dental University. The MTT Cell Proliferation Assay Kit (Cayman Chemical, Ann Arbor, MI, USA) was used to the optimal concentration of Ce6-MnNPs. Cells at 2.5 × 10^4^ cells/mL were incubated with different concentrations of the Ce6-MnNPs (0, 0.1, 0.5, 1.0 and 10 ng/µL) for 2 h and replaced with new culture medium overnight. After adding 10 µL of MTT reagent and 100 µL of crystal dissolving solution to each well, the absorbance of each sample was measured at 570 nm using a microplate reader. Similarly, cells were treated with different light intensities (0.1 W, 0.5 W, 1.0 W; Watt: W) or irradiation times (10 s, 20 s, 40 s, 60 s), and mitochondrial activity was detected by the method described above to select the most suitable treatment conditions. Mitochondrial activity was calculated in the following manner: control group (no treatment) as 100%, mitochondrial activity = 100 × experiment group’s absorbance/control group’s absorbance × 100%.

### 2.6. Near-Infrared Light Photodynamic Therapy

HSC-3 was incubated with Ce6-MnNPs for two hours. Cells were then exposed to a light dose of 10 J/s (0.5 W × 20 s) from a semiconductor laser with a wavelength of 980 nm (Wuhan Pioon Technology, Wuhan, China).

### 2.7. Detection of Change in Mitochondrial Membrane Potential (MMP)

The MMP was examined with a JC-1 MitoMP Detection Kit (Dojindo Laboratories, Kumamoto, Japan). Cells were seeded at 5 × 10^3^ cells/mL on a 96-well plate (black well and clear bottom; Greiner Bio-One, Tokyo, Japan) and incubated for 24 h. After NIR-PDT, the fluorescence change was analyzed using a microplate reader (red: Ex 535 nm and Em 585–605 nm; green: Ex 485 nm and Em 525–545 nm).

### 2.8. Apoptotic Assay

An Annexin V-FITC apoptosis kit (Nacalai Tesque, Kyoto, Japan) was used to detect cell apoptosis after treatment with NIR-PDT, according to the manufacturer’s instructions. Data acquisition and analysis were carried out using confocal laser scanning microscopy (LSM700; Carl Zeiss, Oberkochen, Germany) and flow cytometry (FACSverse; BD Biosciences, San Jose, CA, USA).

### 2.9. Real-Time Quantitative PCR (RT-qPCR)

About 1 × 10^6^ HSC-3 cells were seeded into a 6-well plate and then treated with NIR-PDT. After washing with PBS, total RNA was extracted using an RNeasy Mini Kit (Qiagen, Hilden, Germany), and single-stranded cDNA was synthesized using ReverTra Ace™ qPCR RT Master Mix (Toyobo, Osaka, Japan). The qPCR reaction was prepared with Thunderbird^TM^ Next SYBR qPCR Mix (Toyobo) and examined with the Step One Plus system (Applied Biosystems, Waltham, MA, USA). The formula 2^−ΔΔCT^ was used for quantifying the RT-qPCR results. The primers used for PCR were as follows: *GAPDH*, 5′-GCATCCTGGGCTACACTGAG-3′ (forward) and 5′-AAAGTGGTCGTTGAGGGCAA-3′ (reverse); *Bax*, 5′-CGGGTTGTCGCCCTTTTCTA-3′ (forward) and 5′-GAAGTCCAATGTCCAGCCCA-3′ (reverse); *Bcl-2*, 5′-CTTTGAGTTCGGTGGGGTCA-3′ (forward) and 5′-ATCCACAGGGCGATGTTGTC-3′ (reverse). Relative gene expression was determined and normalized to *GAPDH* expression.

### 2.10. Immunofluorescence Staining

The cells were seeded at 5 × 10^3^ cells/mL on an 8-well slide and chamber (Watson Bio Lab, San Diego, CA, USA). After NIR-PDT, the cells were fixed with 10% formalin (Fujifilm Wako Pure Chemical). After PBS washing, the cells were osmotically treated with 0.25% Triton X-100 (Sigma-Aldrich Corp., St. Louis, MO, USA) for 30 min at room temperature. After being fixed with 1% bovine serum albumin (BSA, Biowest, Nuaillé, France) for 30 min at room temperature, the cells were incubated with primary antibody p53 (DO-7; Dako, Glostrup, Denmark; diluted 1:100) overnight at 4 °C. The secondary antibody used was polyclonal rabbit anti-mouse immunoglobulins/FITC (Dako; diluted 1:500). After washing with PBS, the cells were mounted in VECTASHIELD mounting medium for detecting the fluorescence of DAPI (Vector Laboratories, Burlingame, CA, USA). Immunostaining was visualized using confocal laser scanning microscopy (LSM700; Carl Zeiss).

### 2.11. Statistical Analysis

Statistical differences between the experimental and control groups were analyzed using Student’s *t*-test. *p*-values were calculated using Excel software, and less than 0.05 was considered statistically significant.

## 3. Results

### 3.1. Characterization of Ce6-MnNPs

**Figure 1:**
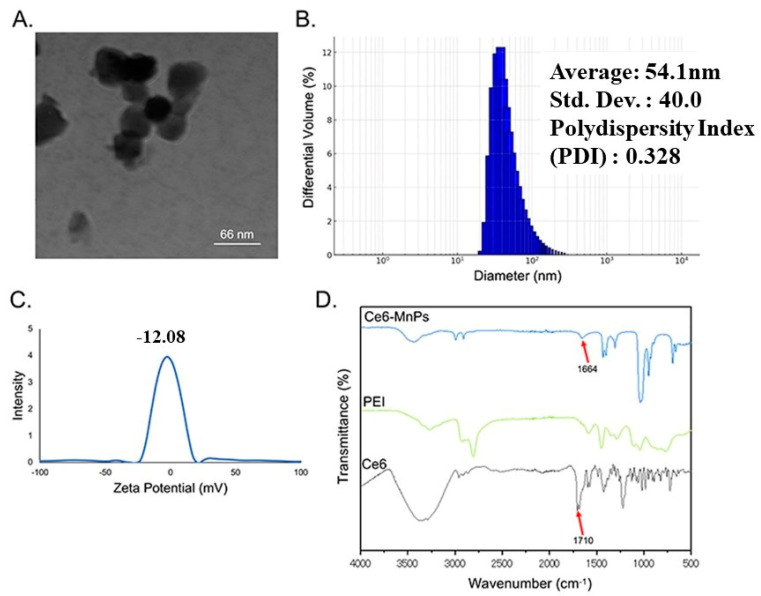
(**A**) TEM image of Ce6−MnNPs. (**B**) Particle size analysis of Ce6−MnNPs. (**C**) Zeta potential of Ce6−MnNPs. (**D**) Fourier−transform infrared spectra of Ce6, PEI and Ce6−MnNPs.

The TEM image (Figure 1A) shows that the size of the synthesized complex is around 55 nm. The results of the particle size distribution (Figure 1B) also show that the size of the complex diameter is concentrated around 50 nm. The mean particle diameter was 54.1 nm, Standard Deviation (S.D.) was 40.0 and the polydispersity index (PDI) was 0.328. The zeta potential showed a negative value of −12.08 mV (Figure 1C). FTIR was used to analyze the chemical structure of Ce6, PEI and photosensitizer complexes. The characteristic functional group of Ce6 is the carboxyl group, where the O-H stretching vibration ranges from 3300 to 2500 cm^−1^ and the C=O stretching vibration ranges from 1720 to 1706 cm^−1^ [20]. The 1664 cm^−1^ absorption peak in the complex appeared as the amide III bond (C=O) stretching vibration (1680–1630 cm^−1^) (Figure 1D) [25]. This is due to the chemical reaction between the carboxyl group of Ce6 and the amino group of PEI. It also indicates the successful combination of up-conversion particle, photosensitizer and PEI.

### 3.2. Ce6-MnNPs Accumulation in HSC-3 Cells

**Figure 2:**
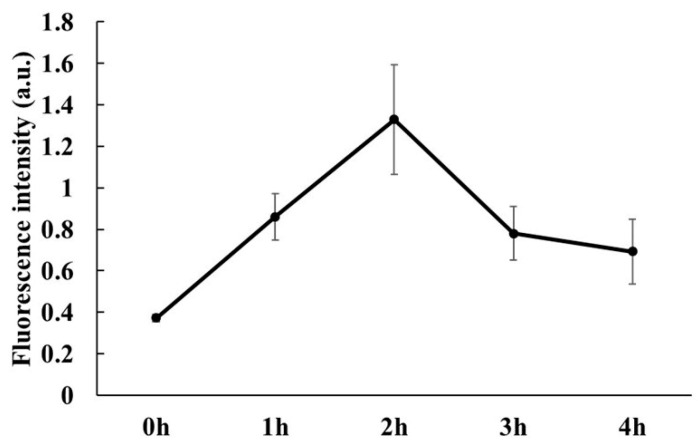
Fluorescence intensity of Ce6 in HSC-3 cells at different incubation times (h: hour).

The detected fluorescence intensity value reflects the accumulation of Ce6-MnNPs in the HSC-3 cell. The value was maximum at 2 h and decreased with increasing incubation time (Figure 2). Therefore, a 2 h incubation time was chosen for the following experiments.

### 3.3. Inhibition of Cell Proliferation

**Figure 3:**
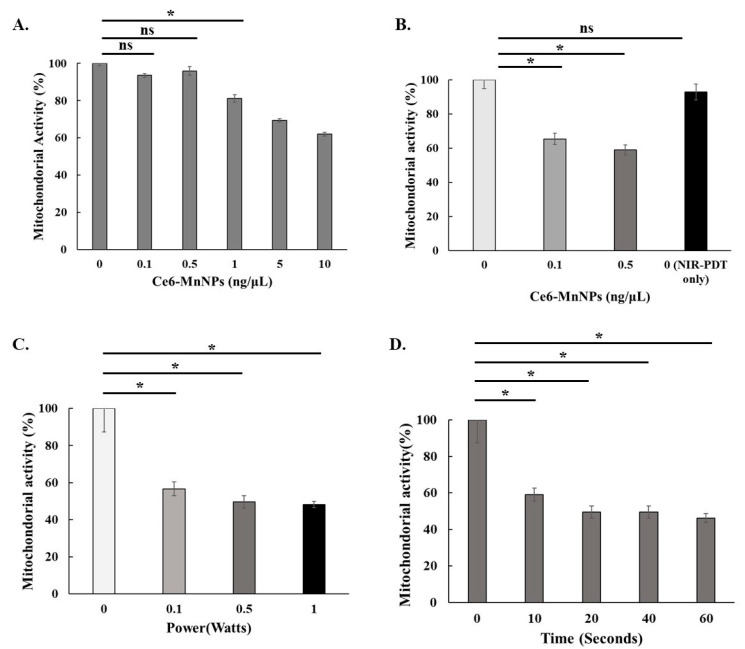
Effect of Ce6-MnNPs and NIR-PDT on cell proliferation for different concentrations of Ce6-MnNPs without NIR-PDT (**A**); with Ce6-MnNPs for different concentrations with NIR-PDT (10J) (**B**); for different irradiation intensities and times (**C**,**D**) (* *p* < 0.05, ns: Not significant).

Concentrations of Ce6-MnNPs at 0.1 and 0.5 ng/µL had no significant effect on the cells (Figure 3A). After treating cells with NIR, the mitochondrial activity rate decreased. However, the laser-only group showed no influence (Figure 3B). In addition, the mitochondrial viability at the same complex concentration was approximately the same at irradiation intensities of 0.5 W and 1.0 W (Figure 3C), and there was no significant decrease after prolonging the irradiation time up to 60 s (Figure 3D). In vitro, NIR-PDT inhibited proliferation with the combination of 0.5 ng/µL Ce6-MnNPs at 0.5 Watts for 20 s.

### 3.4. NIR-PDT Induced Apoptosis in HSC-3 Cells

**Figure 4:**
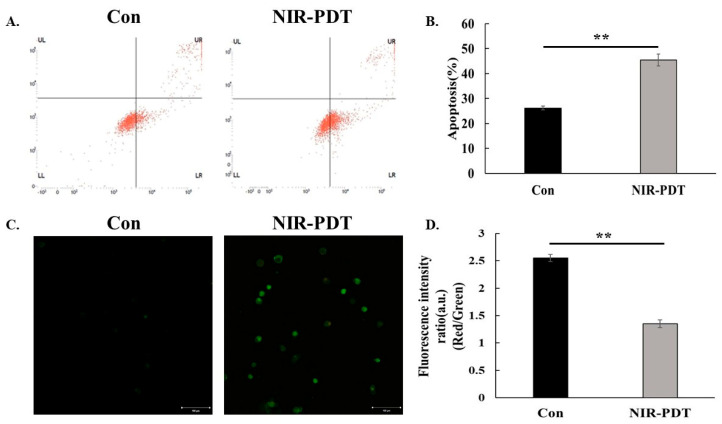
(**A**–**C**) Apoptotic cells were analyzed by Annexin V−FITC/PI kit. (**D**) Mitochondrial membrane potential changes in the HSC−3 cell. (Control: No treatment; NIR−PDT: NIR−PDT+Ce6−MnNPs, Bar: 100 µm, ** *p* < 0.01).

Apoptosis was analyzed by Annexin V-FITC/PI staining. Compared with the control group (FITC and PI negative), the apoptotic rate increased after treatment and was positive for both FITC and PI (Figure 4A). As shown in Figure 4B, there was a significant difference between the NIR-PDT group and control group. Also, in the experimental group we observed apoptotic cells showing green fluorescence by fluorescence microscopy (Figure 4C). The change of mitochondrial membrane potential from high to low also indicates the occurrence of apoptosis, which is manifested as a decrease in the ratio of red fluorescence to green fluorescence (Figure 4D).

**Figure 5:**
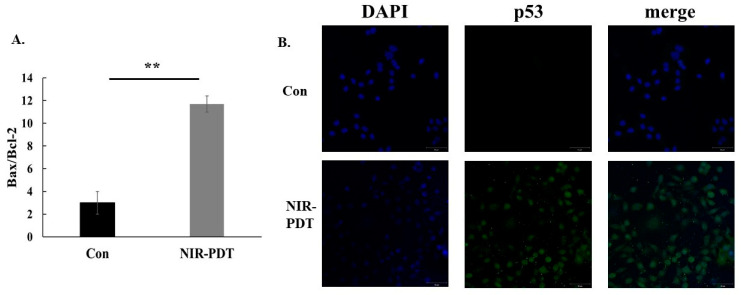
(**A**) The relative expression of mRNA for Bax and Bcl-2 by RT-qPCR. (**B**) HSC-3 was stained with p53 (green) and DAPI (blue). (Control: No treatment; NIR-PDT: NIR-PDT+Ce6-MnNPs, Bar: 50 µm, ** *p* < 0.01).

To further confirm that the NIR-PDT induced apoptosis, RT-qPCR analysis of mRNA was performed. Relative to the control group, the mRNA levels of the NIR-PDT group showed that the ratio of Bax/Bcl-2 increased (Figure 5A). According to the immunofluorescence staining, there was also an increase in p53-positive cells that showed green fluorescence after NIR-PDT (Figure 5B).

## 4. Discussion

In addition to curing the disease, the treatment of malignant tumors should also focus on the quality of the patient’s subsequent survival [26]. Photodynamic therapy, as a new type of alternative therapy with the advantages of a small invasive surface and targeting, has been widely used in the treatment of various diseases [27]. However, there are still many factors that affect the therapeutic effect, for example, the stability of photosensitizers, limited tissue penetration, and so on [28,29]. To overcome these problems, photosensitizers carrying nanoparticles have emerged. In this study, the results of FTIR spectroscopy showed that the amide peaks observed in the synthesized nanoparticles indicate that the amino group of the PEI encapsulating the nanoparticles is bonded to the carboxyl group of Ce6. PEI on nanoparticles is generally known to support stability and cellular uptake, and the presence of amino groups in the synthesized complexes gives good bioactivity [30,31]. The TEM showed an average diameter of about 55 nm; according to Gustafson HH et al., this size of nanoparticles is just right for uptake by cellular endocytosis mediated by small concave proteins [32]. Furthermore, the smaller the particle size, the better the drug delivery and absorption. A dispersivity index describing the particle size distribution greater than 0.5 indicates a wide distribution (0.328 in the present result), but of course a range of 0.1–0.25 is more likely to achieve the desired goals [33]. Measurement of zeta potential also indicated that the complex was stable [34]. Factors such as the raw material used for synthesis and the presence of hydrophobic/hydrophilic and other functional groups in the complex itself can also affect the cellular uptake [35].

After determining the incubation time and concentration, the therapy was shown to have an effect on the proliferation of HSC-3. In photodynamic therapy, apoptosis, necrosis and autophagy are common modes of cell death [36]. In this study, we focused on the relevant indexes of apoptosis. The occurrence of apoptosis is often accompanied by nuclear condensation, DNA fragmentation and the formation of apoptotic vesicles [37]. The detection kit employed here, Annexin-V-FITC and propidium iodide (PI), is used for the detection of apoptosis and necrosis in a wide range of cells. When apoptosis occurs, intracellular phosphatidylserine is exposed on the membrane surface and is recognized by Annexin-V to produce green fluorescence. Only the cell membrane is damaged, and the permeability changes so that PI can enter, eventually showing red fluorescence [38]. In flow cytometry and fluorescence microscopy, apoptosis appeared in HSC-3 after light exposure.

Disruption of mitochondrial activity is also a feature of early apoptosis. This includes changes in membrane potential. The JC-1 stain, which naturally exhibits green fluorescence, is widely used in apoptosis studies in a variety of cells. Under normal conditions, it enters and accumulates in negatively-charged mitochondria, forming red fluorescent aggregates. In apoptotic cells, the mitochondrial membrane potential is lost, the negative charge decreases, and JC-1 accumulates to a lesser extent, retaining its original green monomeric form. Therefore, the ratio of red to green fluorescence intensity directly reflects this apoptotic change [39]. After detecting the changes in mitochondrial membrane potential, the membrane potential of the experimental group was found to be lower than that of the control group. Bcl-2 family proteins are also closely linked to mitochondria when apoptosis occurs [40], and so we examined the expression of the relevant mRNA and the ratio of classical pro-apoptotic Bax and inhibitory apoptotic Bcl-2. The results showed that the ratio was increased in the experimental group. In healthy cells, Bax exists as a monomer in the endoplasmic reticulum, and when apoptosis occurs, it is transferred to the mitochondria [41,42]. Deficiency of Bax results in the blockage of behaviors downstream of apoptosis in the mitochondrial pathway, such as cytochrome c release, loss of mitochondrial membrane potential and chromatin breaks [41]. The opposite is true for Bcl-2, which protects the release of cytochrome c from mitochondria, thereby inhibiting apoptosis and attenuating the therapeutic effect of PDT [43]. Thus, the ratio of Bax to Bcl-2 reflects the efficacy.

p53 is a transcription factor encoded by the TP53 gene on human chromosome 17. Under physiological conditions, various stress responses, such as UV and IR exposure, activate the p53 signaling pathway in cells, which suppresses tumor growth by inducing cell cycle arrest, immune response, apoptosis and the maintenance of genome stability [44,45,46]. It has been shown that PDT induces p53 accumulation in cancer cells and is associated with apoptosis. For example, Wu et al. reported that PDT induced p53 accumulation in colon and breast cancer cells [47,48]. In our experiment, the expression of cellular p53 was observed by immunofluorescence staining after light exposure. Figure 5B shows an increase in positive cells with green fluorescence, while most of the cells in the control group showed blue fluorescence of DAPI. HSC-3 cells with low expression of p53 showed enhanced expression after light exposure. In addition, gain-of-function mutant p53 can inhibit the expression of keratin 17, a diagnostic and prognostic marker for OSCC, according to Enaka et al. [49]. The research has also shown that apoptosis or growth arrest can be induced by transfection of wild-type p53 plasmids in gene therapy for cancer [46]. However, mutations in p53 have a potential impact on its function, with 75% of p53 mutations leading to loss of p53 function and exerting opposite effects, such as promoting invasion, proliferation, cell survival, cancer progression and metastasis [44]. The relationship between PDT and p53, as well as the associated molecular signaling pathways, are next in line for research. That is, the increase in p53 expression is a good trend after treatment.

## 5. Conclusions

**Figure 6:**
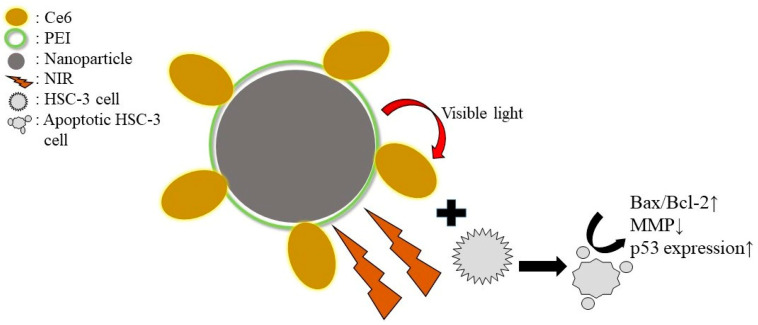
Synthesized nanoparticle photosensitizer complex induces apoptosis in HSC-3 cells under NIR-PDT (↑: The ratio increased and the expression was up-regulated. ↓: Potential decreased).

In the present study, irradiation with NIR in combination with a synthesized nanoparticle photosensitizer complex inhibited oral cancer cell proliferation to some extent by inducing apoptosis production. The expression of the oncogene p53 was also up-regulated (Figure 6), which further confirmed the anti-cancer effect of the method.

However, there are some inevitable problems with such complexes, including the tendency to aggregate and high production costs, which may affect their targeted therapy and widespread use in the clinic. This requires further research and precise design to improve the efficacy and safety of such complexes, and ultimately to realize the full scale-up of the treatment of malignant diseases in the oral cavity and other areas.

## Figures and Tables

**Table 1:** Chemical reagents required for synthesis.

Chemical Reagent Name	Purchasing Company
yttrium(III) chloride hexahydrate (YCl_3_•H_2_O)	Sigma-Aldrich, Tokyo, Japan
N-hydroxysuccinimide (NHS)	Sigma-Aldrich, Tokyo, Japan
ytterbium(III) chloride hexahydrate (YbCl_3_•6H_2_O)	Fujifilm Wako Pure Chemical, Osaka, Japan
erbium(III) chloride (ErCl_3_•6H_2_O)	Fujifilm Wako Pure Chemical, Osaka, Japan
manganese(II) chloride tetrahydrate (MnCl_2_•4H_2_O)	Fujifilm Wako Pure Chemical, Osaka, Japan
polyethyleneimine (PEI; Mw = 1800)	Fujifilm Wako Pure Chemical, Osaka, Japan
ethylene glycol (EG)	Tokyo Chemical Industry, Tokyo, Japan
Ammonium fluoride (NH_4_F)	Tokyo Chemical Industry, Tokyo, Japan

## Data Availability

The original contributions presented in the study are included in the article, further inquiries can be directed to the corresponding author.
